# Improved Stability Criteria of Static Recurrent Neural Networks with a Time-Varying Delay

**DOI:** 10.1155/2014/391282

**Published:** 2014-02-24

**Authors:** Lei Ding, Hong-Bing Zeng, Wei Wang, Fei Yu

**Affiliations:** ^1^School of Information Science and Engineering, Jishou University, Jishou 416000, China; ^2^School of Electrical and Information Engineering, Hunan University of Technology, Zhuzhou 412007, China; ^3^Hunan Railway Professional Technology College, Zhuzhou 412001, China; ^4^Jiangsu Provincial Key Laboratory for Computer Information Processing Technology, Soochow University, Soochow 215006, China

## Abstract

This paper investigates the stability of static recurrent neural networks (SRNNs) with a time-varying delay. Based on the complete delay-decomposing approach and quadratic separation framework, a novel Lyapunov-Krasovskii functional is constructed. By employing a reciprocally convex technique to consider the relationship between the time-varying delay and its varying interval, some improved delay-dependent stability conditions are presented in terms of linear matrix inequalities (LMIs). Finally, a numerical example is provided to show the merits and the effectiveness of the proposed methods.

## 1. Introduction

During the past decades, recurrent neural network (RNN) has been successfully applied in many fields, such as signal processing, pattern classification, associative memory design, and optimization. Therefore, the study of RNN has attracted considerable attention and various issues of neural networks have been investigated (see, e.g., [1–4] and the references therein). As the integration and communication delay is unavoidably encountered in implementation of RNN and is often the main source of instability and oscillations, much efforts have been expended on the problem of stability of RNNs with time delays (see, e.g., [5–14]).

RNNs can be classified as local field networks and static neural networks based on the difference of basic variables (local field states or neuron states) [15]. Recently, the stability of static recurrent neural networks (SRNNs) with time-varying delay was investigated in [16], where sufficient conditions were obtained guaranteeing the global asymptotic stability of the neural network. Nevertheless, some negative semi-definite terms were ignored in [16], which lead to the conservatism of the derived result. By retaining these terms and considering the low bound of the delay, some improved stability conditions were derived for SRNNs with interval time-varying delay in [17]. In [18], an input-output framework was proposed to investigate the stability of SRNNs with linear fractional uncertainties and delays. Based on the augmented Lyapunov-Krasovskii functional approach, some new conditions were derived to assure the stability of SRNNs in [19–22], but the results can be further improved.

In this paper, the problem of stability of SRNNs with time-varying delay is investigated based on the complete delay-decomposing approach [12]. By employing a reciprocally convex technique, some sufficient conditions are derived in the forms of linear matrix inequalities (LMIs). The effectiveness and the merit are illustrated by a numerical example.

*Notations*. Through this paper, *N* ^*T*^ and *N* ^−1^ stand for the transpose and the inverse of the matrix *N*, respectively; *P* > 0  (*P* ≥ 0) means that the matrix *P* is symmetric and positive definite (semipositive definite); ℝ^*n*^ denotes the *n*-dimensional Euclidean space; diag⁡{⋯} denotes a block-diagonal matrix; ||*z*|| is the Euclidean norm of *z*; the symbol ∗ within a matrix represents the symmetric terms of the matrix; for example, $\left[\begin{array}{cc} X & Y\\ {}* & Z \end{array}\right]=\left[\begin{array}{cc} X & Y\\ Y^{T} & Z \end{array}\right]$. Matrices, if not explicitly stated, are assumed to have compatible dimensions.

## 2. System Description

Consider the following delayed neural network:

(1) $$\begin{array}{c} \dot{x}\left(t\right)=-Ax\left(t\right)+f\left(Wx\left(t-\tau \left(t\right)\right)+J\right),\\ x\left(t\right)=\phi \left(t\right),\;-\bar{\tau }\leq t\leq 0, \end{array}$$

where *x*(*t*) = [*x*_1_(*t*), *x*_2_(*t*),…, *x*_*n*_(*t*)]^*T*^ ∈ ℝ^*n*^ and *J* = [*j*_1_, *j*_2_,…, *j*_*n*_]^*T*^ ∈ ℝ^*n*^ denote the neuron state vector and the input vector, respectively; *f*(·) = [*f*_1_(·), *f*_2_(·),…, *f*_*n*_(·)]^*T*^ ∈ ℝ^*n*^ is the neuron activation function; *ϕ*(*t*) is the initial condition; *A* = diag⁡(*a*_1_, *a*_2_,…, *a*_*n*_) > 0 and *W* are known interconnection weight matrices; and *τ*(*t*) is the time-varying delay and satisfies

(2) $$\begin{array}{c} 0\leq \tau \left(t\right)\leq \bar{\tau }, \end{array}$$

(3) $$\begin{array}{c} \dot{\tau }\left(t\right)\leq \mu . \end{array}$$

Furthermore, the neuron activation functions satisfy the following assumption.

**Assumption 1**

The neuron activation functions are bounded and satisfy

(4) $$\begin{array}{c} 0\leq \frac{f_{i}\left(\alpha_{1}\right)-f_{i}\left(\alpha_{2}\right)}{\alpha_{1}-\alpha_{2}}\leq l_{i},\;\forall \alpha_{1},\alpha_{2}\in \mathbb{R}, \end{array}$$

where *l*_*i*_ ≥ 0 for *i* ∈ 1,2,…, *n*. For simplicity, denote *L* = diag⁡(*l*_1_, *l*_2_,…, *l*_*n*_).

Under Assumption 1, there exists an equilibrium x* of (1). Hence, by the transformation z* = *x*(·) − x*, (1) can be transformed into

(5) $$\begin{array}{c} \dot{z}\left(t\right)=-Az\left(t\right)+g\left(Wz\left(t-\tau \left(t\right)\right)\right),\\ z\left(t\right)=\psi \left(t\right),\;-\bar{\tau }\leq t\leq 0, \end{array}$$

where *z*(*t*) = [*z*_1_(*t*), *z*_2_(*t*),…, *z*_*n*_(*t*)]^*T*^ is the state vector; *ψ*(*t*) = *ϕ*(*t*) − x* is the initial condition; and the transformed neuron activation functions *g*(W*z*(·)) = *f*(*Wz*(·) + Wx* + *J*) − *f*(Wx* + *J*) satisfies

(6) $$\begin{array}{c} 0\leq \frac{g_{i}\left(\alpha \right)}{\alpha }\leq l_{i},\;\forall \alpha \neq 0;\;\;g_{i}\left(0\right)=0,\;\;i\in 1,2,\ldots ,n. \end{array}$$

Notice that there exists an equilibrium point *z*(*t*) ≡ 0 in neural network (5), corresponding to the initial condition *ψ*(*t*) ≡ 0. Based on the analysis above, the problem of analyzing the stability of system (1) at equilibrium is changed into a problem of analyzing the zero stability of system (5).

Before presenting our main results, we first introduce two lemmas, which are useful in the stability analysis of the considered neural network.

**Lemma 2 (see [23])**

Let *M* = *M* ^*T*^ > 0 be a constant real *n* × *n* matrix, and suppose $\dot{x}:[-h,0]\mapsto {\mathbb{R}}^{n}$ with *h* > 0 such that the subsequent integration is well defined. Then, one has

(7) $$\begin{array}{c} -h\overset{t}{\underset{t-h}{\int }}{\dot{x}}^{T}\left(s\right)M\dot{x}\left(s\right)ds\leq \zeta^{T}\left(t\right)\left[\begin{array}{cc} -M & M\\ {}* & -M \end{array}\right]\zeta \left(t\right), \end{array}$$

where *ζ*(*t*) = col {*x*(*t*), *x*(*t* − *h*)}.

**Lemma 3 (see [24])**

Let *H*_1_, *H*_2_,…, *H*_*N*_ : ℝ^*n*^ ↦ ℝ be given finite functions, and they have positive values for arbitrary value of independent variable in an open subset *M* of ℝ^*n*^. The reciprocally convex combination of *H*_*i*_  (*i* = 1,2,…, *N*) in *M* satisfies

(8) $$\begin{array}{c} \min\overset{N}{\underset{i=1}{\sum }}\frac{1}{\lambda_{i}}H_{i}\left(t\right)=\overset{N}{\underset{i=1}{\sum }}H_{i}\left(t\right)+\max\overset{N}{\underset{i=1}{\sum }}\overset{N}{\underset{j=1,j\neq i}{\sum }}G_{i,j}\left(t\right) \end{array}$$

subject to

(9) {λi>0,  ∑i=1Nλi=1,  Gi,j(t):ℝn↦ℝ, Gj,i(t)=Gi,j(t),  [Hi(t)Gi,j(t)Gi,j(t)Hj(t)]≥0}.

## 3. Main Results

In the sequel, following the method proposed in [13], we decompose the delay interval $\left[0,\bar{\tau }\right]$ into *m* equidistant subintervals, where *m* is a given integer; that is, $[0,\bar{\tau }]={⋃}_{j=1}^{m}\left[(j-1)\delta ,j\delta \right]$ with $\delta =\bar{\tau }/m$. Thus, for any *t* ≥ 0, there should exist an integer *k* ∈ {1,2,…, *m*}, such that *τ*(*t*) ∈ [(*k* − 1)*δ*, *kδ*]. Then the Lyapunov-Krasovskii functional candidate is chosen as

(10) $$\begin{array}{c} {V(z_{t})|}_{k}:={V(z_{t})|}_{\tau (t)\in [(k-1)\delta ,k\delta ]},\\ {V(z_{t})|}_{k}=V_{1}\left(z_{t}\right)+V_{2}\left(z_{t}\right)+V_{3}\left(z_{t}\right)+V_{4}\left(z_{t}\right)+V_{5}\left(z_{t}\right) \end{array}$$

with

(11) V1(zt)=zT(t)Pz(t)+2∑i=1ndi∫0Wiz(t)gi(α)dα,V2(zt)=∫t−δtζ1T(s)Raζ1(s)ds,V3(zt)=∑j=1mδ∫−jδ−(j−1)δ∫t+θtz˙T(s)Zjz˙(s)ds dθ,V4(zt)=∑j=1k−1∫t−jδt−(j−1)δζ2T(s)𝒬jζ2(s)ds+∫t−τ(t)t−(k−1)δζ2T(s)𝒬kζ2(s)ds,V5(zt)=∑j=1m∫t−jδt−(j−1)δgT(Wz(s))Mjg(Wz(s))ds,

where

(12) $$\begin{array}{c} P>0,\;D=\mathrm{diag}\left\{d_{1},d_{2},\ldots ,d_{n}\right\}\geq 0,\\ R_{a}=\left[\begin{array}{cccc} R_{11} & R_{12} & \cdots & R_{1m}\\ {}* & R_{22} & \cdots & R_{2m}\\ {}* & * & \ddots & \vdots \\ {}* & * & * & R_{mm} \end{array}\right]>0,\;{𝒬}_{j}=\left[\begin{array}{cc} Q_{j} & X_{j}\\ {}* & Y_{j} \end{array}\right]\geq 0,\\ Z_{j}>0,\;M_{j}>0,\;\;j=1,2,\ldots ,m, \end{array}$$

are to be determined, $\zeta_{1}(s)={\left[\begin{array}{cccc} z^{T}(s) & z^{T}(s-\delta ) & \cdots & z^{T}(s-(m-1)\delta ) \end{array}\right]}^{T}$,  $\zeta_{2}(s)={\left[\begin{array}{cc} z^{T}(s) & g^{T}(Wz(s)) \end{array}\right]}^{T}$, and *W*_*i*_  denotes the *i*th row of matrix *W*.

**Remark 4**

Notice that a novel term *V*_4_(*x*_*t*_) being continuous at *τ*(*t*) = *τ*_*k*_ is included in the Lyapunov-Krasovskii functional (10), which plays an important role in reducing conservativeness of the derived result.

Next, we develop some new delay-dependent stability criteria for the delayed neural networks described by (5) and (6) with *τ*(*t*) satisfying (2) and (3). By employing the Lyapunov-Krasovskii functional (10), the following theorem is obtained.

**Theorem 5**

For a given positive integer *m*, scalars $\bar{\tau }>0$ and *μ*, the origin of system (5) with the activation function satisfying (6) and a time-varying delay satisfying conditions (3) is globally asymptotically stable if there exist

(13) $$\begin{array}{c} P>0,\;R_{a}=\left[\begin{array}{cccc} R_{11} & R_{12} & \cdots & R_{1m}\\ {}* & R_{22} & \cdots & R_{2m}\\ {}* & * & \ddots & \vdots \\ {}* & * & * & R_{mm} \end{array}\right]>0,\\ {𝒬}_{j}=\left[\begin{array}{cc} Q_{j} & X_{j}\\ {}* & Y_{j} \end{array}\right]\geq 0,\;Z_{j}>0,\;M_{j}>0,\;j=1,2,\ldots ,m, \end{array}$$

and *D* = diag⁡{*d*_1_, *d*_2_,…, *d*_*n*_} ≥ 0, *T*_1_ = diag⁡{*t*_11_, *t*_12_,…, *t*_1*n*_} ≥ 0, *T*_2_ = diag⁡{*t*_21_, *t*_22_,…, *t*_2*n*_} ≥ 0, and *G*_*j*_, *j* = 1,2,…, *m*, with appropriate dimensions, such that, for *k* = 1,2,…, *m*,

(14) $$\begin{array}{c} \left[\begin{array}{ccc} \Omega_{11}^{\left(k\right)} & \Omega_{12}^{\left(k\right)} & \delta_{1}\Gamma_{1}^{T}\bar{Z}\\ {}* & \Omega_{22}^{\left(k\right)} & \delta_{1}\Gamma_{2}^{T}\bar{Z}\\ {}* & * & -\bar{Z} \end{array}\right]<0, \end{array}$$

(15) $$\begin{array}{c} \left[\begin{array}{cc} Z_{k} & G_{k}\\ {}* & Z_{k} \end{array}\right]>0, \end{array}$$

where

(16) Ω11(k)=Φ11+Φ¯11(k)+Λ1(k),Ω12(k)=Φ12+Λ2(k),Ω22(k)=Φ22+Λ3(k),Φ11 =[0000⋯00∗φ11φ12φ13⋯φ1m0∗∗φ22φ23⋯φ2m−R1m∗∗∗φ33⋯⋮⋮∗∗∗∗⋱φ(m−1)m−R(m−2)m∗∗∗∗⋯φmmZm−R(m−1)m∗∗∗∗⋯∗−Zm−Rmm],Φ12=[WTLT200⋯0PW00⋯0000⋯0⋮⋮⋮⋱⋮000⋯0],Φ22=[β1WTD⋯00∗β2⋯00⋮⋮⋱⋮⋮∗∗⋯βm+10∗∗⋯∗βm+2],Φ¯11(k)=(ψ¯ij(k))(m+2)×(m+2)+(ψ¯ij(k))(m+2)×(m+2)T,Λ1(k)=diag⁡{Λ11(k),Λ22(k),…,Λ2(m+2)(k)},Λ2(k)=diag⁡{Λ21(k),Λ12(k),…,Λ1(m+2)(k)},Λ3(k)=diag⁡{Λ31(k),Λ32(k),…,Λ3(m+2)(k)},Γ1=[0−A0⋯0],Γ2=[I0⋯0],Z¯=∑j=1mZj

with

(17) φij={PA+ATP+R11−Z1,i=j=1,R12+Z1,i=1, j=2,Rij,i=1, 3≤j≤m,Rij−R(i−1)(j−1)−Zi−Zi−1,i=j=2,3,…,m,Rij−R(i−1)(j−1)+Zi,2≤i≤m−1, j=i+1,Rij−R(i−1)(j−1),otherwise,βj={−2T2,j=1,−2T1+M1,j=2,Mj−1−Mj−2,3≤j≤m+1,−Mm,j=m+2,ψ¯ij(k)={−Zk+Gk,i=j=1,Zk−GkT,i=1,j=k+1,Zk−Gk,i=1,j=k+2,−Zk+Gk,i=k+1,j=k+2,0,otherwise,Λ1j(k)={−(1−μ)Qk,j=1,Q1,j=2,Qj−1−Qj−2,3≤j≤k+1,0,otherwise,Λ2j(k)={−(1−μ)Xk,j=1,X1+WTLT1−ATWTD,j=2,Xj−1−Xj−2,3≤j≤k+1,0,otherwise,Λ3j(k)={−(1−μ)Yk,j=1,Y1,j=2,Yj−1−Yj−2,3≤j≤k+1,0,otherwise.

**Proof**

From Assumption 1, it can be deduced that, for any diagonal matrices *T*_*i*_ ≥ 0,  *i* = 1,2,

(18) 0≤2gT(Wz(t))T1[LWz(t)−g(Wz(t))],0≤2gT(Wz(t−τ(t)))×T2[LWz(t−τ(t))−g(Wz(t−τ(t)))].

Now, calculating the derivative of *V*(*z*_*t*_)|_*k*_ along the solutions of neural network (5) yields

(19) $$\begin{array}{c} {\dot{V}(z_{t})|}_{k}={\dot{V}}_{1}\left(z_{t}\right)+{\dot{V}}_{2}\left(z_{t}\right)+{\dot{V}}_{3}\left(z_{t}\right)+{\dot{V}}_{4}\left(z_{t}\right), \end{array}$$

where

(20) V˙1(zt)=2zT(t)Pz˙(t)+2gT(Wz(t))DWz˙(t),V˙2(zt)=ζ1T(t)Raζ1(t)−ζ1T(t−δ)Raζ1(t−δ),V˙3(zt)=∑j=1m{δ2z˙T(t)Zjz˙(t)−δ∫t−jδt−(j−1)δz˙T(s)Zjz˙(s)ds},V˙4(zt)=∑j=1k−1ξT(t−jδ)(𝒬j+1−𝒬j)ξ(t−jδ)+ξT(t)𝒬1ξ(t)−(1−τ˙(t))ξT(t−τ(t))𝒬kξ(t−τ(t))≤∑j=1k−1ξT(t−jδ)(𝒬j+1−𝒬j)ξ(t−jδ)ξT(t)𝒬1ξ(t)−(1−μ)ξT(t−τ(t))𝒬kξ(t−τ(t)),V˙5(zt)=∑j=1m−1gT(Wz(t−jδ))(Mj+1−Mj)g(Wz(t−jδ))+gT(Wz(t))M1g(Wz(t))−gT(Wz(t−τ¯))Mmg(Wz(t−τ¯)).

By Lemmas 2 and 3, it can be deduced that

(21) −δ∫t−kδt−(k−1)δx˙T(s)Zkx˙(s)ds =−δ∫t−τ(t)t−(k−1)δx˙T(s)Zkx˙(s)ds    −δ∫t−kδt−τ(t)x˙T(s)Zkx˙(s)ds ≤−δτ(t)−(k−1)δ[x(t−(k−1)δ)x(t−τ(t))]T[Zk−Zk∗Zk]  ×[x(t−(k−1)δ)x(t−τ(t))]  −δkδ−τ(t)[x(t−τ(t))x(t−kδ)]T[Zk−Zk∗Zk][x(t−τ(t))x(t−kδ)] ≤−[x(t−(k−1)δ)−x(t−τ(t))x(t−τ(t))−x(t−kδ)]T[ZkGk∗Zk]  ×[x(t−(k−1)δ)−x(t−τ(t))x(t−τ(t))−x(t−kδ)] =ξT(t)[−2Zk+Gk+GkTZk−GkTZk−Gk∗−ZkGk∗∗−Zk]ξ(t),

where *ξ* ^*T*^ = [*x*(*t* − *τ*(*t*))^*T*^ *x*(*t* − (*k* − 1)*δ*)^*T*^ *x*(*t* − *kδ*)^*T*^].

Next, we introduce a new vector as

(22) $$\begin{array}{c} \zeta \left(t\right)={\left[\begin{array}{cc} \zeta_{1}^{T}\left(t\right) & \zeta_{2}^{T}\left(t\right) \end{array}\right]}^{T}, \end{array}$$

where

(23) $$\begin{array}{c} \zeta_{1}\left(t\right)=\left[\begin{array}{c} z\left(t-\tau \left(t\right)\right)\\ z\left(t\right)\\ z\left(t-\delta \right)\\ z\left(t-2\delta \right)\\ \vdots \\ z\left(t-m\delta \right) \end{array}\right],\;\zeta_{2}\left(t\right)=\left[\begin{array}{c} g\left(Wz\left(t-\tau \left(t\right)\right)\right)\\ g\left(Wz\left(t\right)\right)\\ g\left(Wz\left(t-\delta \right)\right)\\ g\left(Wz\left(t-2\delta \right)\right)\\ \vdots \\ g\left(Wz\left(t-m\delta \right)\right) \end{array}\right]. \end{array}$$

Then, rewrite system (5) as

(24) $$\begin{array}{c} \dot{z}\left(t\right)=\left[\begin{array}{cc} \Gamma_{1} & \Gamma_{2} \end{array}\right]\zeta \left(t\right). \end{array}$$

Adding the right sides of (18) to (19) and applying (21) yield

(25) $$\begin{array}{c} {\dot{V}(z_{t})|}_{k}=\zeta {\left(t\right)}^{T}\left(t\right)\left[\Omega^{\left(k\right)}+\delta^{2}\Gamma^{T}\overset{m}{\underset{j=1}{\sum }}Z_{j}\Gamma \right]\zeta \left(t\right), \end{array}$$

where

(26) $$\begin{array}{c} {\bar{\Omega }}^{\left(k\right)}=\left[\begin{array}{cc} \Omega_{11}^{\left(k\right)} & \Omega_{12}^{\left(k\right)}\\ {}* & \Omega_{22}^{\left(k\right)} \end{array}\right],\\ \Gamma =\left[\begin{array}{cc} \Gamma_{1} & \Gamma_{2} \end{array}\right]. \end{array}$$

For all *k* = 1,…, *m*, if *Ω* ^(*k*)^ + *δ* ^2^Γ^*T*^∑_*j*=1_^*m*^ *Z*_*j*_Γ < 0, which is equivalent to LMIs (14) in the sense of Schur complement [25], then ${\dot{V}(z_{t})|}_{k}<0$ for any *ζ*(*t*) ≠ 0. Note that *V*(*z*_*t*_) is continuous at *τ*(*t*) = *τ*_*k*_, so the system (5) is globally asymptotically stable. This completes the proof.

**Remark 6**

In the proof of Theorem 5, *τ*(*t*)−(*k* − 1)*δ* and *kδ* − *τ*(*t*) are not simply enlarged to *δ* as [16] does. By employing reciprocally convex approach to consider this information, Theorem 5 may be less conservative, which will be verified by the simulation results in the next section.

**Remark**

In previous works such as [16, 19], considerable attention has been paid to the case that the derivative of the time-varying delay $\dot{\tau }(t)$ satisfies (3). However, in the case of $\dot{\tau }(t)$ satifying

(27) $$\begin{array}{c} \dot{\tau }\left(t\right)\leq \mu_{k},\;\tau \left(t\right)\in \left[\tau_{k-1},\tau_{k}\right],\;k=1,2,\ldots ,m, \end{array}$$

the treatment in [16, 19] means that $\dot{\tau }(t)$ in (27) is enlarged to $\dot{\tau }(t)\leq \mu =\max\{\mu_{1},\mu_{2},\ldots ,\mu_{m}\}$, which may lead to conservativeness inevitably. By contrast, the case above can be taken fully into account by replacing *μ* with *μ*_*k*_ in Theorem 5.

For the case that the time-varying delay *τ*(*t*) is nondifferentiable or $\dot{\tau }(t)$ is unknown, setting 𝒬_*j*_ = 0,  *j* = 1,2,…, *m*, in Theorem 5, a delay-dependent and rate-independent criterion is easily derived as follows.

**Corollary 8**

For a given positive integer *m*, scalars $\bar{\tau }>0$, the origin of system (5) with the activation function satisfying (6) and a time-varying delay satisfying condition (2) is globally asymptotically stable if there exist

(28) $$\begin{array}{c} P>0,\;R_{a}=\left[\begin{array}{cccc} R_{11} & R_{12} & \cdots & R_{1m}\\ {}* & R_{22} & \cdots & R_{2m}\\ {}* & * & \ddots & \vdots \\ {}* & * & * & R_{mm} \end{array}\right]>0,\\ Z_{j}>0,\;M_{j}>0,\;j=1,2,\ldots ,m,\\ D=\mathrm{diag}\left\{d_{1},d_{2},\ldots ,d_{n}\right\}\geq 0,\\ T_{1}=\mathrm{diag}\left\{t_{11},t_{12},\ldots ,t_{1n}\right\}\geq 0,\\ T_{2}=\mathrm{diag}\left\{t_{21},t_{22},\ldots ,t_{2n}\right\}\geq 0,\\ G_{j},\;j=1,2,\ldots ,m, \end{array}$$

with appropriate dimensions, such that, for *k* = 1,2,…, *m*, LMIs in (15) and (29) hold

(29) $$\begin{array}{c} \left[\begin{array}{ccc} \Phi_{11}+{\bar{\Phi }}_{11}^{\left(k\right)} & \Phi_{21} & \delta_{1}\Gamma_{1}^{T}\bar{Z}\\ {}* & \Phi_{22} & \delta_{1}\Gamma_{2}^{T}\bar{Z}\\ {}* & * & -\bar{Z} \end{array}\right]<0, \end{array}$$

where $\Phi_{11},\;\;\Phi_{12},\;\;\Phi_{22},\;\;{\bar{\Phi }}_{11}^{\left(k\right)},\;\;\Gamma_{1}$, and Γ_2_ are defined in Theorem 5.

## 4. Numerical Examples

In this section, we will provide a numerical example to show the effectiveness of the presented criteria.

**Example 1**

Consider neural network (1) with the following parameters:

(30) $$\begin{array}{c} A=\left[\begin{array}{ccc} 7.3458 & 0 & 0\\ 0 & 6.9987 & 0\\ 0 & 0 & 5.5949 \end{array}\right],\\ W=\left[\begin{array}{ccc} 13.6014 & -2.9616 & -0.6936\\ 7.4736 & 21.6810 & 3.2100\\ 0.7920 & -2.6334 & -20.1300 \end{array}\right]. \end{array}$$

The activation functions satisfy (6) with

(31) $$\begin{array}{c} L=\mathrm{diag}\left(0.3680,0.1795,0.2876\right). \end{array}$$

This example has been discussed in [16–22]. By using Theorem 5 and Corollary 8 with *m* = 2, for various *μ*, the upper bounds $\bar{\tau }$ that guarantee the global asymptotic stability of neural network (1) are computed and listed in Table 1. It can be concluded that the upper bounds obtained by our method are much better than those in [16–22]. Obviously, the conditions proposed in this paper are an improvement over the existing ones.

## 5. Conclusions

This paper has studied the stability of SRNNs by constructing a complete delay-decomposing Lyapunov-Krasovskii functional. Some improved delay-dependent stability conditions have been derived by utilizing a reciprocally convex technique to consider the relationship between the time-varying delay and its varying interval, which are formulated in linear matrix inequalities (LMIs). Finally, a numerical example has been provided to show the effectiveness of the proposed methods.

## Figures and Tables

**Table 1:** Allowable upper bounds of $\overset{-}{\tau }$ for different *μ*.

*μ*	0	0.1	0.5	0.9	Any *μ*
[16]	1.3323	0.8245	0.3733	0.2343	0.2313
[19]	1.3325	0.8404	0.4265	0.3217	0.3211
[20]	1.3324	0.8402	0.4266	0.3225	0.3218
[17]	1.3323	0.8402	0.4264	0.3214	0.3209
[18] (*N* = 1)	1.5157	0.9279	0.4267	—	0.3212
[18] (*N* = 2)	1.5330	0.9331	0.4268	—	0.3215
[21]	—	0.8411	0.4267	0.3227	0.3215
[22]	1.5575	0.9430	0.4417	0.3632	0.3632

The proposed (*m* = 2)	1.7685	1.0431	0.4382	0.3668	0.3644
